# Novel experimental setup for time-of-flight mass spectrometry ion detection in collisions of anionic species with neutral gas-phase molecular targets

**DOI:** 10.1140/epjti/s40485-015-0023-9

**Published:** 2015-08-21

**Authors:** J C Oller, L. Ellis-Gibbings, F. Ferreira da Silva, P. Limão-Vieira, G. García

**Affiliations:** Instituto de Física Fundamental, Consejo Superior de Investigaciones Científicas, Serrano 113-bis, 28006 Madrid, Spain; Centro de Investigaciones Energéticas, Medioambientales y Tecnológicas, Avenida Complutense 22, 28040 Madrid, Spain; Laboratório de Colisões Atómicas e Moleculares, CEFITEC, Departamento de Física, Faculdade de Ciências e Tecnologia, Universidade Nova de Lisboa, 2829-516, Caparica, Portugal; Centre of Medical Radiation Physics, University of Wollongong, Wollongong, NSW 2522 Australia

**Keywords:** Hollow cathode discharge, Anion beam, Collisions, TOF, Negative ions, Atomic and molecular collisions, Electron transfer

## Abstract

We report a novel experimental setup for studying collision induced products resulting from the interaction of anionic beams with a neutral gas-phase molecular target. The precursor projectile was admitted into vacuum through a commercial pulsed valve, with the anionic beam produced in a hollow cathode discharge-induced plasma, and guided to the interaction region by a set of deflecting plates where it was made to interact with the target beam. Depending on the collision energy regime, negative and positive species can be formed in the collision region and ions were time-of-flight (TOF) mass-analysed. Here, we present data on O_2_ precursor projectile, where we show clear evidence of O^–^ and O_2_^–^ formation from the hollow cathode source as well as preliminary results on the interaction of these anions with nitromethane, CH_3_NO_2_. The negative ions formed in such collisions were analysed using time-of-flight mass spectrometry. The five most dominant product anions were assigned to H^–^, O^–^, NO^–^, CNO^–^ and CH_3_NO_2_^–^.

## Introduction

The study of radiation interactions with key biological constituents at the molecular level, has shown an increasing interest in the last few years, in particular after the pioneering studies of Sanche and co-workers on the resonant formation of DNA strand breaks by low-energy electrons [1, 2]. Such level of interactions has generated in the international community an urgent need to explore the different underlying molecular mechanisms responsible for such modifications, because mutagenic and genotoxic effects have been identified to be closely related to the initial molecular alterations. A comprehensive description of these mechanisms may ultimately lead to the development of new strategies and protocols in cancer/radiation therapy.

New radiotherapy techniques based on ion beam irradiation and using nanoparticles as radiosensitizers, concentrate the energy deposition around reduced volumes where, abundant secondary species, e.g. electrons and radicals, are generated. These secondary species have been found to be more efficient in producing damage than the primary radiation, because they can trigger physicochemical processes which determine the radiation damage in terms of molecular structure alterations (e.g. bond breaking, ionisation and negative ion formation, just to mention a few). In this context, event by event Monte Carlo simulation codes [3] together with multiscale modelling procedures [4] have been recently developed in order to model the effect of the radiation in reduced volumes and its correlation with the observed damage. Improving the accuracy of these models requires a considerable amount of interaction data that must be obtained from experiments and theory. In particular, interaction probabilities for ionic and neutral radicals generated during the irradiation are scarce, and in most cases almost unknown. Many elementary collisional processes are not due to direct electron impact but rather depend upon electron transfer, either from neutral [5] or even anionic species [6]. These conditions motivated the present work in which a novel experimental system has been developed to study the interaction of negative radical species with biological relevant molecular targets. The essential part of this setup is composed of a pulsed hollow cathode discharge source, where upon gas admission allows extracting negative species to form a collimated beam with selected energies, in the range (0-1000 eV), to interact with an effusive molecular target. Ionic species, either positive or negative, formed in the interaction region are time-of-flight (TOF) mass analysed, although in this contribution we focus our attention to anion formation only.

In the next section we present a description of the novel experimental set up, and in section 3 we discuss the preliminary set of results obtained with O_2_ feed gas discharge and negative ion formation with nitromethane, CH_3_NO_2_ molecules. Finally, in section 4, some concluding remarks from this investigation are summarized.

### Experimental details

**Fig. 1:**
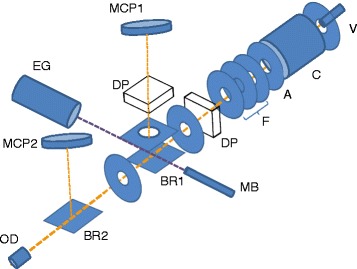
Schematics of the experimental setup. V, pulsed supersonic valve; C, hollow cathode discharge; A, anode; F, focusing lens; DP, deflecting plates; BR, beam reflector; MB, effusive molecular beam; MCP, multi-channel plate detector; EG, electron gun; OD, optical detector

**Fig. 2:**
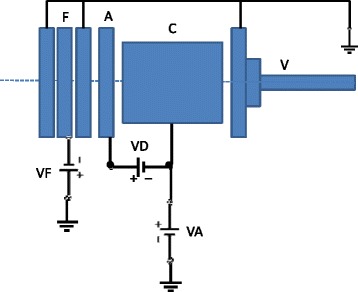
Electrical schematics of the anion beam source. V, pulsed supersonic valve; C, hollow cathode discharge; A, anode; F, focusing lens; VD, discharge voltage; VA, accelerating voltage; VF, focusing voltage

The experimental setup has been developed at CSIC Madrid, consisting mainly of two interconnected high-vacuum chambers (projectile and collision chambers), both differentially pumped reaching an ultimate base pressure of 1.2 × 10^-8^ mbar, equipped with a hollow cathode discharge source, a linear time-of-flight (TOF) mass spectrometer, a set of deflecting and focusing plates, an electron gun and multi-channel plate detectors (see Fig. 1). The precursor gas projectile was admitted into vacuum through a commercial Parker pulse valve (VAC1250) [7], where the base pressure increased to 4 × 10^-8^ mbar, and made to cross at right angles with an effusive molecular beam, with a base pressure up to 1–2 × 10^-7^ mbar in the collision chamber. The anionic beam was formed in a hollow cathode discharge-induced plasma at a negative voltage (0–1000 V) relative to the anode, with the cathode also assembled on a positive voltage (0–1000 V) relative to ground (Fig. 2). Such arrangement allows varying the anionic species energy, permitting therefore to explore the collision dynamics. The pulse valve typically operates at 340 μs width in 80 ms duty-cycle. After the hollow cathode source, a set of three element einzel lens and XY deflecting plates are mounted so that the former arrangement allows to improve the anionic beam focus, whilst the latter allows to correct for any deflections (Fig. 1). With such arrangement, the anionic beam can be made to reach the interaction region ensuring the highest intensity for the collision process. The target molecule is brought to gas-phase by a 0.5 mm diameter capillary to yield an effusive molecular beam of a gas or liquid, or in the case of solid samples through a resistive-heated oven.

The anionic and neutral molecular beams interact within a collision region, where a pulsed electrostatic field of −600 V, 0-8 μs variable width, 80 ms duty-cycle and 1500 μs delayed from the anionic pulse beam, is applied between two parallel plates (BR1 in Fig. 1) at a mutual distance of 15 mm. Negative ions formed in the interaction region through the electron transfer process, are removed in a direction normal to the plane of the two crossing beams. The electric field pushes the resulting negative ions into a 1.12 m TOF mass spectrometer, where anions are mass analysed and detected in a microchannel plate (MCP1) working on single pulse count mode (Fig. 1). However, if no collision is performed (and so no extraction is applied), the anionic projectile beam can be probed by a microchannel plate (MCP2) placed 5 cm just above the plane normal to its initial main direction at 0.65 m from the hollow cathode source (Fig. 1). Here a negative voltage of – 700 V is applied to BR2 (see Fig. 1). As shown in Fig. 1(a), the negative ion signal is formed during a short period of time in the afterglow, once plasma generated species de-excited and secondary electron attachment processes are favourable. This MCP2 detector is also used to monitor the alignment of the primary anion beam. In both set of measurements, a high-resolution digital oscilloscope (Tektronix MSO 3034, 2.5GS/s) is used to perform data acquisition.

Additionally, the discharge intensity can be monitored through an optical detector (digital camera), placed just in the beam’s forward direction (Fig. 1). The interaction region is also equipped with a small electron gun to analyse the neutral molecular beam composition and perform electron impact ionization measurements if required.

**Fig. 3:**
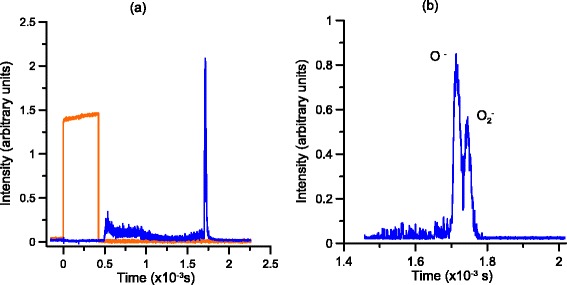
Time analysis of the negative ion beam. **a** Anion signal *blue* with respect to the valve control signal *yellow*. **b** Detail of the negative ion beam composition

**Fig. 4:**
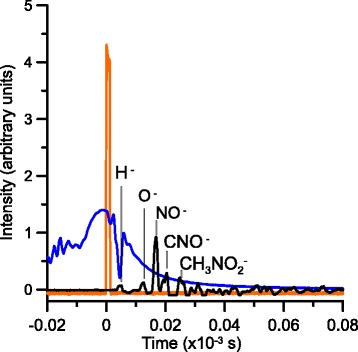
Time analysis of the fragmentation induced to nitromethane molecules by electron transfer from oxygen anions. *Yellow* Beam reflector signal; *blue* primary anion beam; *black* anion induced fragments to nithromethane

The negative ion signals in Figs. 3 and 4 are obtained through TOF mass spectrometry, where the pulse valve is used as a “start” and the resulting signal from the MCP detectors as the “stop”. Furthermore, when running the experimental setup in the TOF mode normal to the plane of the crossing beams, i.e. for negative ion formation in electron transfer from the anionic projectile to the neutral target molecule, the pulsed electrostatic field is set to an adjusted delay in respect to the pulse valve. In the current experiments such corresponds typically to 1500 μs delay. TOF mass assignment is performed on the basis of the well-known anionic spectrum from neutral potassium-nitromethane collision experiments [8].

### Nitromethane samples

The liquid sample of nitromethane, CH_3_NO_2_, was purchased from Sigma-Aldrich with a minimum purity of ≥ 96 % and degassed by a repeated freeze–pump–thaw cycle before admission to the collision chamber.

## Results and discussion

Figure 3 shows the TOF mass spectra of the anions clearly formed in the hollow cathode discharge-induced plasma at –250 V, with O_2_ as the feed gas. In Fig. 3a), and at shorter flight times, the signal also contains contributions from electrons and discharge instability from the hollow cathode source, albeit not interfering at all with that from the anions. The figure shows also the pulse valve control signal to set the proper TOF scale. The anionic beam is comprised of O^–^ and O_2_^–^ anions (see Fig. 3b), the latter yielding around 70 % of the former, and clearly visible from the weak background signal (~10 %). Although both neutral species show a rather modest electron affinity values, EA(O) = 1.439 eV and EA(O_2_) = 0.448 eV [9], they are stable enough within the collision time scale to perform electron transfer experiments yielding anionic species from a neutral target molecule.

In the collision process from the anionic projectile to the neutral target beam, the potential role of the neutral atom as a stabilizing agent (third body) post-electron transfer from the incident anion is of particular interest, when compared to similar experiments performed with O^–^ although the latter at higher impact energies, i.e. 4 keV [6]. While in the current experiments the collision energy of the projectile is well-above that used in neutral alkali atom–molecule collision experiments, comparisons with the potassium projectile [8, 10, 11], and also with electron attachment experiments [12], can shed some light on our understanding of such stabilization effects. Nonetheless the electron capture mechanism, electron transfer from the projectile to the molecule, results in a transient negative ion (TNI) formation, which can decay via electron auto-detachment or fragmentation of the precursor anion.

Figure 4 shows the time analysis of the anionic yields produced from the electron transfer in collisions of oxygen anions (O^–^/O_2_^–^) to nitromethane molecules, CH_3_NO_2_, at 250 eV. In this figure we see the starting pulse that pushes the negative ions into the TOF normal to the plane of the two crossing beams, together with the projectile anionic signal from MCP2 (blue curve). Of relevance, the fact that this signal is suddenly attenuated when the negative extraction voltage is applied, meaning that this voltage is enough to deviate the primary beam. However, the projectile’s kinetic energy is high enough to avoid any focusing within the TOF tube, even at a moderate extraction voltage to be detected in the MCP1, i.e. these anions are certainly lost in collisions with the TOF tube and do not reach the MCP detector. Moreover, the lack of any O_2_^–^ signal means that the anionic pattern in Fig. 4 is due to negative ions from CH_3_NO_2_ only.

Recently, nitromethane anion formation has been comprehensively studied in the gas-phase by dissociative electron attachment (DEA) [12] and electron transfer in neutral alkali atom collisions [9]. Nitromethane has a sufficiently large dipole moment, 3.46 D, above the critical value of ∼ 2.5D [13] to bind an extra electron in a stable dipole-bound state. In DEA experiments, the dominant fragment anion was NO_2_^−^ and no parent anion, CH_3_NO_2_^−^, was detected. This is reasonable due to the small positive electron affinity of nitromethane (0.44 eV). In the present study, we observe that the major anionic fragments from the collision of O^–^/O_2_^–^ with nitromethane, are assigned to NO^–^ and CNO^–^, with other minor contributions from anionic signals assigned to H^–^, O^–^ and the parent anion CH_3_NO_2_^–^. This is in contrast to previous O^−^ collision experiments at 4 keV with nitromethane, where the parent anion CH_3_NO_2_^–^ was the dominant signal [6] and other less intense fragments assigned to NO_2_^−^ and H^−^. As a comparison, results obtained in neutral alkali atom collision experiments have shown that the main fragment was due to NO_2_^−^ but, in contrast to DEA experiments, parent anion CH_3_NO_2_^–^ formation was observed. From Fig. 4, NO and CNO anion formation seems reasonable due to their high electron affinity values, ~3.0 and 3.6 eV [9], respectively, whereas no clear evidence for NO_2_^–^ formation is observed. However, due to lack of proper mass (time) resolution, we note that the CNO^–^ peak may accommodate a second structure which could be easily assigned to NO_2_^–^. Such certainly needs to be properly explored in future investigations when mass resolution issues will be appropriately addressed.

Nitromethane’s stable dipole-bound anionic state, provides a doorway into valence states of the molecular parent anion [14, 15]. The dipole-bound molecular anion has a significantly different geometry from its neutral counterpart, where a symmetric bend of the oxygen atoms in the –NO_2_ group results in a tetrahedral shape [16]. Similarly to both alkali atom collisions and Rydberg electron transfer experiments, nitromethane parent anion formation by negative ion impact is expected to proceed through a transition to a low vibrational state of a ^2^*B*_1_ anionic state [17], whereas this does not occur in free electron attachment interactions. Another rationale to support such parent anion formation relies on the interaction of the projectile electron donor and the molecular target, modifying therefore the relative position or shape of the potential surfaces, and thereby changing the dissociation pathways. Owing to the high dipole moment of nitromethane, the presence of O/O_2_ in the collision complex may be rationalised as a third body “forcing” the electron to remain in the dipole-bound state long enough for the molecule to adiabatically proceed into its anionic geometry, thereby allowing an intramolecular electron transfer into one of its valence orbitals (in the anionic geometry). This is in contrast with free electron interactions where, even if the electron is initially captured into a dipole-bound state, its lifetime is not long enough to compete with auto-detachment. For a comprehensive description of the lowest-lying anionic states of nitromethane that may be involved, see [6, 12] and references therein.

Nonetheless, we observe significant differences in the anionic yields of nitromethane against O^–^ experiments at 4 keV [6], and these need to be proper explored in future work which is far beyond the context of this contribution.

## Conclusions

We report a novel experimental setup for studying collision induced products resulting from the interaction of anionic beams with a neutral gas-phase molecular target. The precursor anionic beam projectile is produced in a hollow cathode discharge-induced plasma, guided to the interaction region by a set of deflecting plates and made to interact with a neutral target beam.

In this work we obtained TOF mass spectra of negative ions produced in the collision of an anion projectile (O^–^/O_2_^–^) with neutral nitromethane molecules. The fragmentation pattern is significantly different from that obtained both in dissociative electron attachment and in alkali atom–molecule collision experiments. It is interesting to note that the absence, or even low yield, of NO_2_^−^, lends credibility to the assumption that the stabilizing effect produced by the potassium cation in neutral alkali atom collisions [8, 17] stems from the electrostatic interaction between the potassium cation and the molecular TNI [8]. This mechanism has also been reported with biological molecules elsewhere [5, 10, 11]. Future comprehensive and detailed work exploring these mechanisms in anion–molecule collisions may provide some answers regarding to the role of such electron transfer processes.
